# Enhanced echovirus 11 genomic surveillance in neonatal infections in Spain following a European alert reveals new recombinant forms linked to severe cases, 2019 to 2023

**DOI:** 10.2807/1560-7917.ES.2024.29.44.2400221

**Published:** 2024-10-31

**Authors:** Maria Dolores Fernandez-Garcia, Nerea Garcia-Ibañez, Juan Camacho, Almudena Gutierrez, Laura Sánchez García, Cristina Calvo, Antonio Moreno-Docón, Ana Isabel Menasalvas, Antonio Medina, Mercedes Perez-Ruiz, Maria Carmen Nieto Toboso, Carmen Muñoz-Almagro, Cristian Launes, Carla Berengua, María Cabrerizo, the Spanish study group for Enterovirus and Parechovirus infections (PI22CIII-00035), The Spanish study group for Enterovirus and Parechovirus infections, Carlos Grasa, Isabel Mellado, Blanca Bravo, Iker Falces Romero, Margarita del Cuerpo, Ana Navascués, Elisa Garrote, Ana Bordes, Eduardo Lagarejos, Gregoria Megias, Juan Valencia-Ramos, Amaresh Pérez-Arguello, Miguel Blanco-Fuertes

**Affiliations:** 1 Enterovirus and Viral Gastroenteritis Unit, National Centre of Microbiology, Instituto de Salud Carlos III, Majadahonda, Madrid, Spain; 2 CIBER Epidemiology and Public Health (CIBERESP), Madrid, Spain; 3 Hospital Universitario La Paz, Madrid, Spain; 4 IdiPaz Foundation, Translational Research Network in Pediatric Infectious Diseases, Hospital Universitario La Paz, Madrid, Spain; 5 CIBER Infectious Diseases (CIBERINFEC), Madrid, Spain; 6 Hospital Clínico Universitario Virgen de la Arrixaca, IMIB-Arrixaca, Murcia University, Murcia, Spain; 7 Hospital Universitario Regional de Málaga, Málaga, Spain; 8 Hospital Universitario de Basurto, Bilbao, Spain; 9 Institut de Recerca Sant Joan de Déu, Hospital Sant Joan de Déu, Barcelona, Spain; 10 Medicine Department, Universitat Internacional de Catalunya, Barcelona, Spain; 11 Hospital de la Santa Creu i Sant Pau, Universitat Autònoma de Barcelona, Barcelona, Spain; 12 The members of the study group are listed under Collaborators

**Keywords:** echovirus 11, Enterovirus, neonatal infection, new variant, recombinant, genomic surveillance, international alert

## Abstract

**Background:**

In 2023, a European alert was issued regarding an increase in severe enterovirus (EV) neonatal infections associated with echovirus 11 (E11) new lineage 1.

**Aim:**

To analyse E11-positive cases between 2019 and 2023 to investigate whether the new lineage 1 circulated in Spain causing severe neonatal infections.

**Methods:**

EV-positive samples from hospitalised cases are sent for typing to the National Reference Enterovirus Laboratory. Available samples from 2022–23 were subjected to metagenomic next-generation sequencing.

**Results:**

Of 1,288 samples genotyped, 103 were E11-positive (98 patients: 6 adults, 33 neonates, 89 children under 6 years; male to female ratio 1.9). E11 detection rate was similar before and after detection of the new lineage 1 in Spain in June 2022 (9.7% in 2019 vs 10.6% in 2023). The proportion of E11-infected ICU-admitted neonates in 2019–2022 (2/7) vs 2022–2023 (5/12) did not significantly differ (p = 0.65). In severe neonatal infections, 4/7 E11 strains were not linked to the new lineage 1. The three novel E11 recombinant genomes were associated with severe (n = 2) and non-severe (n = 1) cases from 2022–2023 and clustered outside the new lineage 1. Coinfecting pathogenic viruses were present in four of 10 E11-positive samples.

**Conclusion:**

The emergence of the new lineage 1 is not linked with an increase in incidence or severity of neonatal E11 infections in Spain. The detection of two novel E11 recombinants associated with severe disease warrants enhancing genomic and clinical surveillance.

**Key public health message**

**What did you want to address in this study and why?**

In 2023, a European alert was issued regarding an increase in severe and fatal enterovirus (EV) neonatal infections associated with echovirus 11 (E11) detected in western and southern European countries. All strains were linked to a new lineage 1 first detected in April 2022. Our aim was to examine if the newly reported lineage 1 is circulating in Spain and is associated with severe neonatal infections.

**What have we learnt from this study?**

We did not observe an increase in the incidence of E11 infections among all typed EVs, nor did we observe difference in the proportion of severe E11 neonatal infections in 2022–2023 compared with 2019–2020. Severe cases observed in 2022–2023 were not exclusively linked to the new lineage 1. Two of the five severe cases observed in 2022–2023 were novel recombinant E11 viruses.

**What are the implications of your findings for public health?**

This study calls for strengthening genomic and clinical surveillance in neonatal E11 infections further. Coinfections should be thoroughly investigated as they could contribute to E11 severe outcomes. The study underscores the need to improve clinical information when requesting sample testing to correctly report on E11 severity association in neonates.

## Introduction

Enteroviruses (EV) are common human viruses associated with diverse clinical syndromes ranging from minor febrile illness to rare but severe and potentially fatal conditions such as aseptic meningitis, paralysis, myocarditis and sepsis-like disease, particularly in newborns [1]. The incidence of EV infection among febrile infants admitted to hospital with systemic infection or infants with suspected sepsis has been reported by different authors to range from 3% to 50% [1,2]. Prematurity, maternal history of EV illness, onset of illness within the first days of life and infecting EV-type, especially B species such as coxsackievirus (CV) B or echovirus 11 (E11), are significant factors associated with severe or even fatal neonatal EV infections [1,3-5].

In 2023, France reported nine severe E11 neonatal infections with liver failure and high mortality rate, with pronounced prevalence in male twins [6]. All E11 sequences associated with severe cases clustered in a new lineage 1 that emerged in 2022 [6] leading to a public health international alert [7,8]. Two cases of severe hepatitis associated with the same lineage 1 were reported during the same period (2023) in Italy [9]. Analysis of the complete genomes demonstrated that all severe and fatal cases were associated with an E11 strain of recombinant origin [6,9]. In July 2023, the European Centre for Disease Prevention and Control appealed for enhancing surveillance regarding severe neonatal E11 infections to assess whether the new lineage 1 first detected in France was associated with more severe disease [7].

Similar to that observed in other European Union (EU) countries, E11 in Spain is among the most commonly notified EV genotypes, mostly affecting children under 3 months of age [10-13]. In this study, we describe the molecular epidemiology and clinical characteristics associated with E11 infections between 2019 and 2023 in Spain. The aim was to investigate whether the newly reported lineage 1 variant emerged and circulated in Spain after the COVID-19 pandemic, causing severe neonatal infections.

## Methods

### Data source

For non-polio enterovirus (NPEV) surveillance, Spanish hospitals can send EV-positive samples (stool, cerebrospinal fluid (CSF), blood, throat swabs or RNA extracts) to the National Reference EV Laboratory (EVL) at the National Centre for Microbiology for type characterisation, voluntarily and at the discretion of clinicians. The EVL receives specimens from hospitalised patients with EV infection and different clinical manifestations, mainly neurological, cutaneous or respiratory. The following data are collected for each sample on the request form: date of birth, sex, clinical information, sampling date and referring laboratory and/or clinician. Data from samples collected between January 2019 and December 2023 were included in the study.

### Analysis of samples and sequences

Typing methods used by the EVL include four RT-nested PCRs on the 3’-VP1 region (specific for species EVA, -B, -C and -D68), as described previously [14,15], followed by Sanger sequencing and Basic Local Alignment Search Tool (BLAST) analysis (https://blast.ncbi.nlm.nih.gov/Blast.cgi). Alignments and phylogenetic trees were generated using ClustalW [16] and the MEGA programme version 10 (http://www.megasoftware.net), respectively. In order to assess the phylogenetic relationships of the E11 sequences, these sequences were analysed together with previously reported E11 strains from multiple countries as well as representative reference sequences available in GenBank [17]. Phylogenetic analyses were carried out with the neighbour-joining method based on the Kimura-2 parameter model. Support for tree nodes was assessed by bootstrap values based on 1,000 replicates. Similarity plot analysis for potential recombination were performed using SimPlot, version 3.5.1 [18]. A 200 nt window moved in 20 nt increments using a Kimura 2-parameter method with a transition-transversion ratio of 2 with 1,000 resampling. All publicly available E11 complete genomes (n = 218) as of July 2024 were included in the recombination analysis.

### Metagenomic next-generation sequencing and data analysis

Sample processing for subsequent metagenomic next-generation sequencing (mNGS) and data analysis are detailed in the Supplementary Material. Briefly, after RNA was extracted from clinical samples, sample library preparation was conducted using the NEBNext Ultra II Directional RNA Library Prep Kit for Illumina with NEBNext Multiplex Oligos for Illumina (New England BioLabs Inc., Ipswich, United States (US)). Target enrichment was performed by hybrid capture using the Twist Comprehensive Viral Research Panel version 2 (Twist Biosciences, San Francisco, US). Enriched libraries were sequenced on an Illumina NextSeq500 (300 Cycles). For sequencing data analysis, an in-house viralrecon pipeline was used (https://github.com/nf-core/viralrecon). The resulting raw reads were analysed for quality using FastQC version 0.11.9 [19] and then trimmed using fastp version 0.20.1 [20]. De novo assemblies of non-host reads were generated using SPAdes version 3.14.0 [21] in metaSPAdes mode. Contigs taxonomic annotation was based on alignment to the National Center for Biotechnology Information’s viral database using BLAST version 2.9.0 + [22]. In addition to the de novo assembly, a reference-based mapping approach was used to obtain viral genomes. Reads were mapped against the E11 reference genome (OQ969170​) using Bowtie2 [23]. For coinfecting viruses, reference genome accession numbers were OR728261, PP625122, KJ492899, OZ035228. SAMtools version 1.14 [24] was used to generate genome mapping statistics and coverage depth. The de novo assemblies and mapped sequences were compared and assessed the full identities of both sequences.

FASTQ files were also processed on the IDseq platform (https://czid.org/) a cloud-based open-source tool for pathogen detection from metagenomic data [25]. Briefly, reads are de novo assembled into contigs using SPAdes [21] and mapped back to the resulting contigs using Bowtie2 version 2.3.4.3 to identify the contig to which they belong. Each contig is aligned to the set of possible accession numbers represented by the BLAST database to improve the specificity of alignments to all the underlying reads. We defined a detection of a virus ‘hit’ as a viral taxon with the highest abundance of reads matching to that taxon in the nucleotide (NT) and non-redundant protein (NR) databases, while being less prevalent in control samples selected for the applied background model. Only viruses detected at ≥ 20 reads per million (rpm) based on nt alignments (NT rpm) were considered positive and included in heatmaps generated using iheatmapr [26]. Read counts were normalised to rpm at 10 million reads to reduce differences due to uneven sequencing depths. The z-score was computed with respect to a background model generated on the basis of RNA-sequencing data derived from control samples. All taxa with a z-score less than 1, contig shorter than 35 base pairs (bp) and rpm of less than 10 were removed from the analysis.

### Confirmatory testing

Conventional RT-PCRs and Sanger sequencing were used to confirm the presence of viral genomes identified by mNGS. Human parechovirus (HPeV) type A (HPeV-A) and EV-D68 were assessed by using RT-PCRs as previously described [15,27].

### Statistical analysis

Quantitative variables were expressed as mean and standard deviations (SD) and qualitative variables as proportions. For the comparison of proportions, a chi-squared test was used with a 95% confidence interval (CI) (http://openepi.com/). P values less than 0.05 were considered significant.

## Results

### Echovirus 11 detection and typing

**Figure 1:**
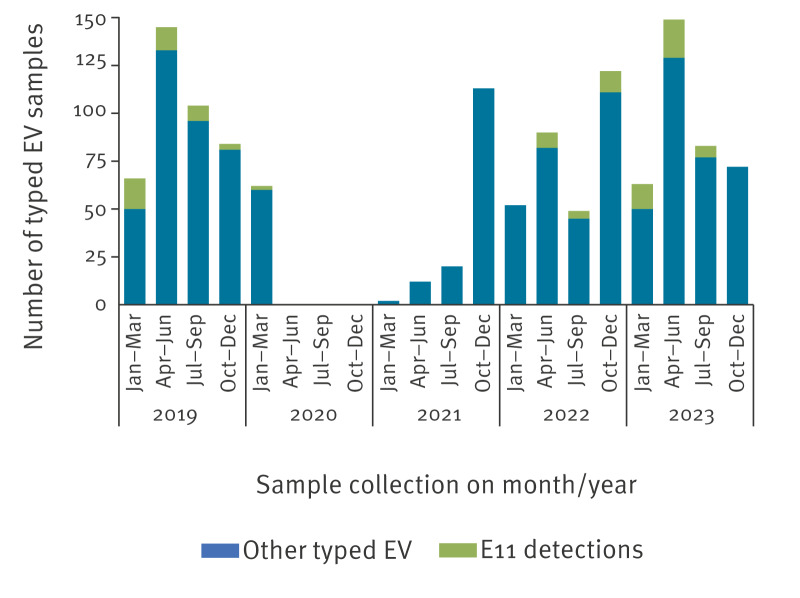
Echovirus 11 detection in enterovirus samples, Spain, January 2019–December 2023 (n = 1,288)

Between January 2019 and December 2023, 1,649 EV-positive samples were received by the EVL for genotyping, as part of the NPEV surveillance. Of these, 1,288 were successfully characterised (78.1%) and a total of 103 EV were identified as E11. All were included in this study and corresponded to 98 patients who attended 36 different hospitals throughout Spain. Echovirus 11 was detected in 2019, 2020 (January–March), 2022 and 2023, accounting for 9.7%, 3.2%, 7.3% and 10.6%, respectively, among all typed EV (Figure 1). In 2021, no E11 were detected. Echovirus 11 was identified in respiratory samples (n = 38, 36.9%), CSF (n = 32, 31.1%), blood (n = 17, 16.5%) and stools (n = 16, 15.1%). In five cases, two E11-positive samples were available for each (four cases had positive CSF and respiratory samples, while one case had two positive faecal samples).

### Clinical and demographic characteristics of echovirus 11-positive patients

Of 98 E11-positive individuals, 65 were male and 33 female (ratio: 1.9, p < 0.001). All but six patients were children, and most of them (89/92, 96.7%) were under 6-years-old with a mean age of 10.3 months (range: 1 day–6 years). The age of the adults ranged from 24 to 90 years. Regarding clinical symptoms, most cases exhibited a febrile syndrome (40 cases, 40.8%). Other clinical symptoms were meningitis/meningoencephalitis (29 cases, 29.6%), respiratory illness (20 cases, 20.4%), encephalitis, ataxia or paralysis (five cases, 5.1%) and neonatal sepsis-like symptoms (four cases, 4.1%).

### Echovirus 11 neonatal infections

Of all E11 infections detected between 2019 and 2023 (n = 98), 33 (33.7%) were in neonates (≤ 28-days-old), 20 males and 13 females (ratio: 1.5, p = 0.06). Mean age of neonates when infected was 15 days (range: 1–28 days). In 21 of the 33 cases, E11 was detected from CSF or blood samples. Echovirus 11-infected neonates with available clinical diagnosis (n = 33) presented febrile syndrome or fever without source (FWS) (14/33), meningitis/meningoencephalitis (13/33), sepsis-like symptoms (4/33) or respiratory illness (2/33) (Table). In five cases, additional symptoms such as irritability, bradypnea syndrome, hypotonia or jaundice were observed. Of the 19 cases with additional clinical data available, seven required admission to an intensive care unit (ICU) and four received treatment (Table). More (n = 12) ICU-admitted neonates were observed between 2022 and 2023 than between 2019 and 2020 (n = 7). No significant difference in the proportion of E11-infected ICU-admitted neonates between the 2019–2020 (2/7) and 2022–2023 (5/12) periods was observed (p = 0.65). The majority (4/7) of ICU-admitted neonates were females. Information on being a twin was available for 19 cases and two male and one female twin pairs were E11-positive.

**Table:** Clinical and demographic characteristics of neonates with a laboratory-confirmed echovirus 11 infection, Spain, 2019–2023

ID	YEAR	Age (days)	Sample type	Clinical diagnosis	Other symptoms	Pre-term	ICU	Death	Treatment	New Lineage 1
SPA19	2019	15–28	CSF	Meningoencephalitis	Fever, respiratory	No	No	No	Oxygen, antibiotics	No
SPA20	2019	15–28	CSF	FWS	NA	NA	NA	NA	NA	No
SPA21	2019	15–28	Blood	Sepsis	Fever	No	Yes	No	Paracetamol	No
SPA22	2019	15–28	CSF	FWS	No	No	No	No	No	No
SPA26	2019	15–28	Stool	FWS	NA	NA	NA	NA	NA	No
SPA31	2019	15–28	CSF	FWS	No	No	No	No	No	No
SPA35	2019	15–28	Throat swab	FWS	NA	NA	NA	NA	NA	No
SPA37	2019	15–28	Throat swab	FWS	NA	NA	NA	NA	NA	No
SPA39	2019	1–14	CSF	Meningoencephalitis	NA	NA	NA	NA	NA	No
SPA40	2019	1–14	Stool	Meningitis	NA	NA	NA	NA	NA	No
SPA41	2019	1–14	Stool	Meningitis	Bradypnea, hypotonia	No	Yes	No	No	No
SPA42	2019	1–14	Throat swab	FWS	NA	NA	NA	NA	NA	No
SPA45	2019	15–28	Throat swab	FWS	NA	NA	NA	NA	NA	No
SPA48	2019	15–28	CSF	FWS	No	No	No	No	No	No
SPA51	2020	1–14	Blood	Respiratory	Fever, irritability	Yes	No	No	No	No
SPA52	2022	1–14	Blood	Sepsis	NA	NA	NA	NA	NA	No
SPA6	2022	15–28	CSF	Meningitis	Fever	No	No	No	No	No
SPA7	2022	1–14	CSF	Meningoencephalitis	Fever, irritability, jaundice	No	Yes	No	No	No
SPA2	2022	15–28	Stool	FWS	No	No	No	No	No	No
SPA71	2022	1–14	CSF	FWS	No	No	No	No	No	Yes
SPA1	2023	1–14	Blood	Sepsis, hepatitis	Liver failure	Yes	Yes	Yes	No	No
SPA3	2023	1–14	Stool	Meningitis	Fever	No	No	No	Cefotaxime	Yes
SPA84	2023	1–14	Blood	FWS	NA	NA	NA	NA	NA	No
SPA86	2023	15–28	Throat swab	Respiratory	Irritability	No	Yes	No	Oxygen, antibiotics	Yes
SPA87	2023	15–28	CSF	Sepsis	Meningitis	NA	NA	NA	NA	Yes
SPA91	2023	1–14	CSF	FWS	No	No	No	No	No	Yes
SPA94	2023	15–28	Throat swab	FWS	Irritability	No	No	No	No	Yes
SPA5	2023	1–14	Stool	Meningoencephalitis	NA	NA	NA	NA	NA	Yes
SPA101	2023	1–14	CSF	Meningitis	NA	NA	NA	NA	NA	Yes
SPA8	2023	15–28	CSF	Meningitis	NA	NA	Yes	No	No	Yes
SPA10	2023	15–28	CSF	Meningitis	NA	NA	Yes	No	No	Yes
SPA112	2023	1–14	Blood	Meningitis	Fever	No	No	No	Paracetamol	Yes
SPA113	2023	15–28	CSF	Meningoencephalitis	NA	NA	NA	NA	NA	Yes

CSF: cerebrospinal fluid; F: female; FWS: fever without a source; ICU: intensive care unit; ID: Identification name; M: male; NA: not available.

The lineage classification was established by phylogenetic analysis based on a 395 bp 3`-VP1 sequence of echovirus 11 strains detected in neonates.

## Phylogenetic analysis of VP1 genomic region

**Figure 2:**
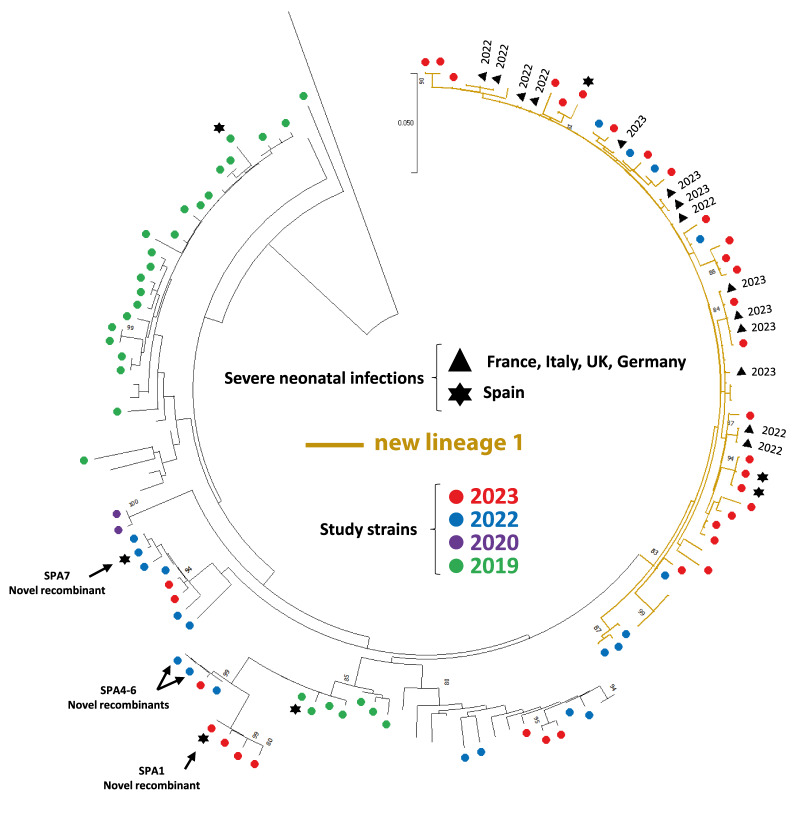
Phylogenetic tree of partial (395 bp) 3´-VP1 coding sequences of 88 echovirus 11 study strains and sequences from previously described echovirus 11 strains extracted from GenBank (n = 46), including new lineage1 variant strains detected in France, Germany Italy, and the United Kingdom, 2013–2023

We performed a phylogenetic analysis with partial VP1 sequences generated in this study (n = 88) and compared the results with VP1 E11 sequences available in GenBank from China, France, Germany, Italy, Spain and the United Kingdom (UK) between 2015 and 2023. A total of 33 E11 study strains (all detected in 2022 and 2023) clustered with the new lineage 1 detected in France, whereas the rest of the sequences were outside the cluster (n = 55, from 2019 to 2023) (Figure 2). The analysis showed that new lineage 1 E11 strains were first detected in Spain in June 2022 (SPA58) details are shown in Supplementary Figure S1. The majority of E11 strains detected in Spain in 2022 and 2023 clustered with the new lineage 1 (61.5% and 71.4%, respectively).

Sequences obtained in this study are indicated by coloured circles with colour according to year of sampling. Phylogenetic analysis was inferred using the neighbour-joining method. Numbers on nodes indicate the bootstrap support of node (> 80). Scale bar represents nt substitutions per site. The echovirus 11 prototype Gregory sequence was used as the outgroup with accession number X80059. A non-collapsed tree can be found in Supplementary Figure S1.

Regarding neonatal infections, 12 of 33 neonates had an E11 strain clustering with the new lineage 1, while the majority (21/33, 63.6%) of neonates were infected by an E11 strain clustering outside the new lineage 1. Considering severe infections as those requiring ICU admission, among the seven E11 strains detected in severe neonatal cases, four did not cluster with the new lineage 1, including the case that resulted in liver failure (SPA1) (Table).

**Figure 3:**
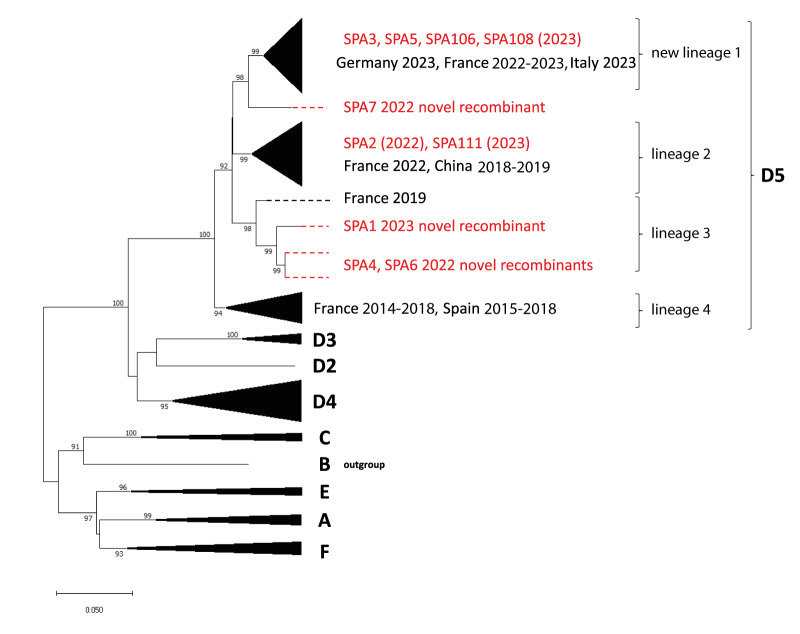
Phylogenetic tree of complete VP1 coding sequences of 10 echovirus 11 study strains and sequences from previously described echovirus 11 strains extracted from GenBank (n = 98), worldwide countries^a^

^a^ For a full list of countries and dates, please see Supplementary Figure S3.

A phylogenetic analysis with the complete VP1 region was performed defining E11 genotypes and sub-genotypes by a nt sequence difference of at least 8% and 15% in the complete VP1 region, respectively [28,29]. All study strains clustered in sub-genotype D5 which diverged into four lineages (Figure 3). Each of these lineages showed less than 8% nt sequence difference and thus cannot consider any of these lineages as a new sub-genotype. Of note, lineage 3 (bootstrap 98%) included only a sequence from France (OR030003, 2019) and sequences from study strains SPA1 (corresponding to a fatal case), SPA4 and SPA6. The phylogeny showed a rapid turnover of D5 lineages over the last years shifting from lineage 4 dominating in 2014–2018 in France and Spain to lineages 1 to 3 dominating in 2022 and 2023 and including fatal and severe neonatal cases [6]. This pattern of clustering was similar to that obtained when the partial VP1 region was used for phylogenetic analysis (Figure 2) with the exception of SPA7. In the complete VP1 (Figure 3) and full genome phylogenetic analyses for which detailed information is available in Supplementary Figure S2, SPA7 clustered closely with the new lineage 1 (bootstraps 99%, and 100%, respectively) in contrast to the partial VP1 analysis. Complete VP1 sequence of SPA7 strain differed from that of other E11 viruses in new lineage 1 by 4%. A notable point is that SPA7 and other similar sequences (SPA54, SPA55, SPA56 and SPA57) were all circulating in Spain in June 2022, coinciding with the first detection of the new lineage 1 E11 study strain (June 2022, SPA58). SPA58 is the strain of new lineage 1 with the closest genetic similarity (96.2%) with SPA7.

Sequences obtained in this study are indicated in red. The tree is annotated with the classification proposed by Li et al. [28]. Phylogenetic analysis was inferred using the neighbour-joining method. Numbers on nodes indicate the bootstrap support of node (> 80). Scale bar represents nt substitutions per site. The echovirus 11 prototype Gregory sequence corresponding to genotype B was used as outgroup (accession number X80059). A non-collapsed tree can be found in Supplementary Figure S3.

### Metagenomic analysis

**Figure 4:**
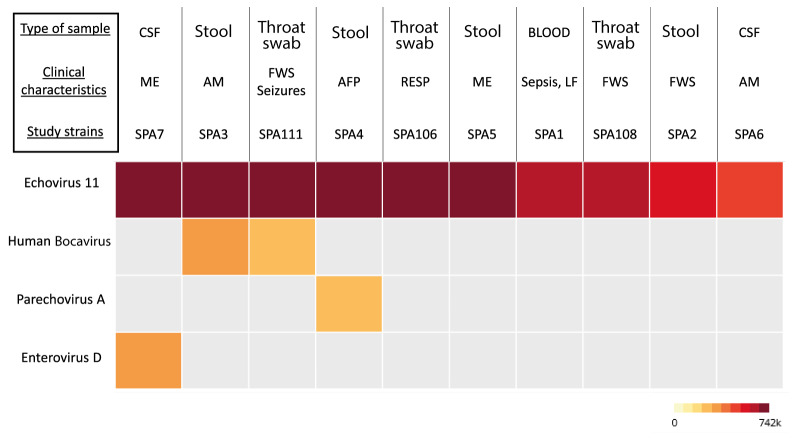
Heatmap analysis showing virus diversity in echovirus 11-positive study samples, Spain, 2022‒2023 (n = 10 samples)

AFP: acute flaccid paralysis; AM: aseptic meningitis; FWS: fever without a source; LF: liver failure; ME: meningoencephalitis; RESP: respiratory disease.

We examined the virome of 16 E11-positive residual samples that tested positive between 2022 and 2023 in Spain and that were available with sufficient volume for mNGS. The full-length genome was successfully obtained for 10 E11 strains. Results of the metagenomic analysis can be found in Supplementary Table S1. In all samples, read mapping to each reference genome was achieved with high coverage of the genome length (> 99%) and sufficient sequencing depth (> 200 for 7/10 sequences and > 40 for 3/10). A heat map was built to analyse the presence of viral reads in each sample and their relative abundance in number of rpm (Figure 4). For all 10 samples, both de novo assembly and reference-based mapping successfully produced complete E11 genomes. Interestingly, in four of these E11-positive samples, reads corresponding to other coinfecting viral pathogens were found by de novo assembly. Human bocavirus was found in two samples and the remaining viruses (HPeV-A and EVD) were single occurrences. Additionally, we performed conventional RT-PCR on the same samples to confirm the presence of the HPeV-A and EV-D68 viral genomes that were identified with mNGS on samples SPA4 and SPA7, respectively. The presence of HPeV-A in sample SPA4 was confirmed by RT-PCR, Sanger sequencing and by reference-based mapping, corresponding to genotype HPeV-A1, as demonstrated in Supplementary Table S1. No positive result was obtained for sample SPA7 after EV-D68 RT-PCR.

Reads per million (rpm) was used to normalise read counts across samples.

Heatmap was generated based on log_10_[rpm]. Coloured logarithmic scale represents rpm, with darker red representing the highest rpm. The heat map was set to NT rpm  ≥  20 which only shows viruses with at least 20 rpm and NT z-score = 100 above background to assure the taxon does not appear in any of the samples in the background model. Each column represents a study sample. Each row represents a virus genus-specific pathogen identified.

### Recombination analysis

Considering recombination is known as a widespread mechanism of EV evolution, we looked for evidence of recombination in the study strains. Whole genome sequences of 10 E11 study strains were compared with publicly available E11 complete genomes from GenBank (n = 105). The phylogenetic analysis using the complete P1 capsid coding region of all these sequences showed that study strains clustered together with the reference genome of prototype strain Gregory (Supplementary Figure S4-A), confirming the preliminary typing results using the partial VP1 in Figure 2. However, this was no longer observed for the nonstructural P2 (Supplementary Figure S4-B) and P3 coding regions (Supplementary Figure S4-C). The P2 and P3-genomic regions of study strains did not cluster with the E11 prototype but rather with other EV-B types or other E11 global strains. These incongruent tree topologies between the structural and nonstructural regions suggested that recombination between E11 study strains and other EV-B types might have occurred.

**Figure 5:**
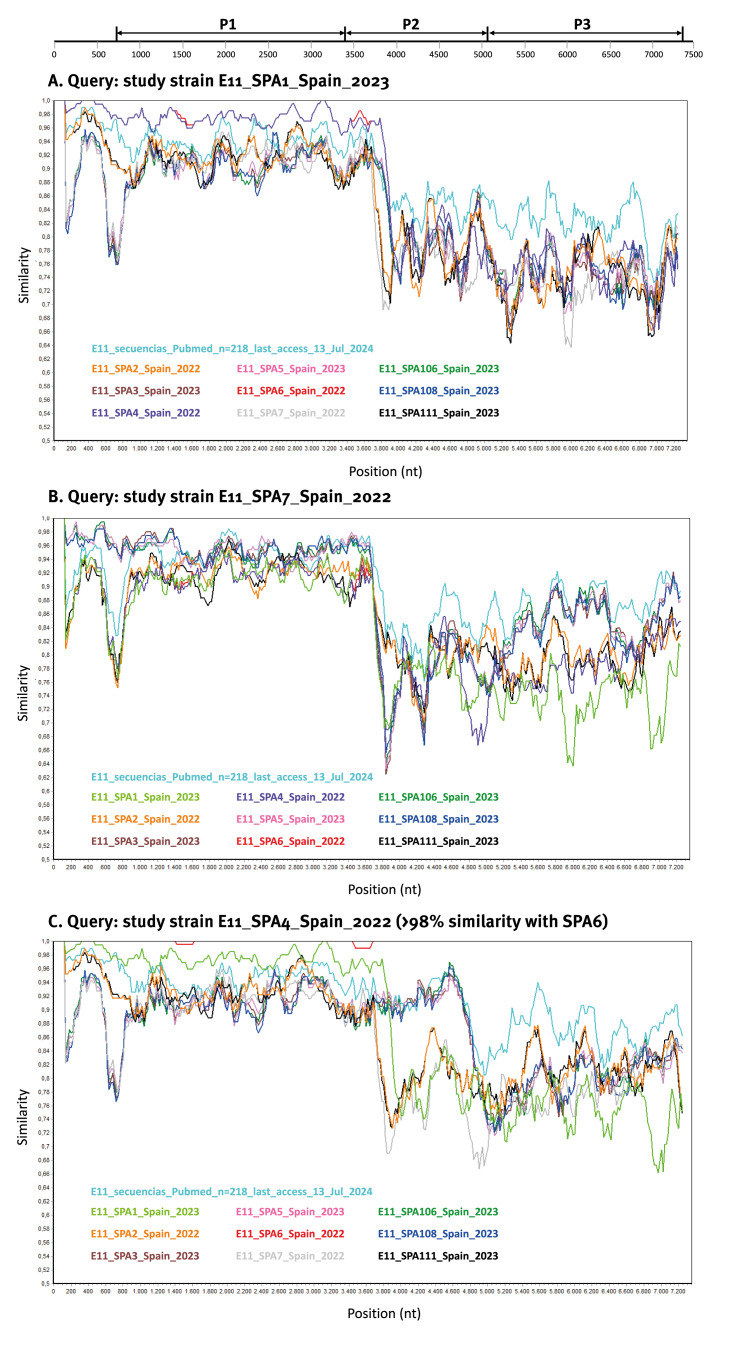
Plot of similarity of whole genome nt sequences of echovirus 11 study strains for (A) query SPA1, (B) query SPA7 and (C) query SPA4 with E11 strains from the National Center for Biotechnology Information

To confirm the existence of recombination events, similarity plot analysis was conducted against all available E11 genomes in GenBank (Figure 5). Regarding the 3′ non-structural half of the genomes, study strains SPA3, SPA5, SPA106 and SPA108 showed high similarity (> 95%) with previously described E11 strains in the new lineage 1 from France (2022–2023), Italy (2023) and Germany (2023), suggesting that their P2-P3 regions share a recent common ancestor (Supplementary Figure S5). Of interest, SPA1 and SPA7 study strains showed a low similarity value (< 90%) in the 3′ half of the genome (P2 + P3) with other E11 strains (Figure 5A, 5B). Similarly, SPA4 and SPA6 (both with > 98% homology between them) showed less than 90% similarity with other E11 strains in the P3 genomic region (Figure 5C). These findings suggest that study strains SPA1, SPA7 and SPA4/SPA6 exhibited a novel recombinant genomic organisation for which no homologous E11 sequences have been detected or reported. For these novel recombinant forms, the highest nt similarity scores in the 3′ half of the genome was lower than 91% with their closest related viruses CV-B4, CV-B5, EV-B79, EV-B80, EV-B88, E4, E13 and E25) indicating a distant genetic relationship (Supplementary Table S2). When analysing the complete VP1 region, the full P1, P2, P3 regions or the full genome, all novel recombinant forms clustered outside the new lineage 1 (Figure 3, Supplementary Figures S4-A, S4-B, S4-C and Supplementary Figure S2) and were associated with fatal sepsis (SPA1), acute flaccid paralysis (SPA4) and meningitis/meningoencephalitis (SPA6/SPA7) cases from 2022 to 2023.

The enterovirus genetic organisation is shown in panel A. Analyses were conducted by using SimPlot 3.5.1 (Kimura distance model, window size 200 bp moving in 20 nt steps).

## Discussion

Our phylogenetic analysis indicates that new lineage 1 E11 strains were first detected in Spain in June 2022 and predominated during 2022 and 2023. The majority of E11 strains detected in Spain during this period clustered with the new lineage 1. This result is in line with a report dating the first detection of this new variant in France in April 2022 [6]. Also, in agreement with the French report, the new lineage 1 included sequences from severe and non-severe neonatal infections as well as non-neonatal infections. However, unlike the French study, not all E11 sequences between 2022 and 2023 that were associated with fatal or severe neonatal infections in Spain were of the new lineage 1, but rather belonged to three different D5 lineages.

Our findings revealed a similar E11 detection rate before and after the first detection of the new lineage 1 in 2022 (9.7% in 2019 vs 10.6% in 2023), excluding the COVID-19 pandemic years 2020–2022. This suggests that, at least in Spain, the emergence in 2022 of the new lineage 1 was not linked with an increased number of E11 infections. Regarding the E11 detection rate in severe neonatal infections, no significant difference in the proportion of ICU-admitted neonates between 2019 and 2020 (2/7) vs 2022–2023 (5/12) was observed. However, our data should be interpreted with caution given the small numbers, and the lack of more comparative clinical data before 2019 given that clinical information is not consistently provided by hospitals when submitting samples. Over 90% of the E11 infections were in children under 4 years of age and, of these, more than one third were in neonates. These results are comparable with that observed in previous studies in Spain and other countries [4,11,30]. Our observation that more than half (57%) of neonates with severe E11 infection were female differed from that of the French and Italian reports where all severe neonatal infections caused by E11 were male [6,9]. Therefore, a hypothetical predisposition to E11 infection associated with the X-chromosome as has previously been suggested, could not be concluded from our data [6].

Phylogenetic analysis indicated that all E11 study strains (2019–2023) clustered in subgenotype D5, which is globally distributed. In Spain, E11 has remained genetically stable, as all sequences analysed between 2002 and 2023 (2019–2023 in this study) clustered together in subgenotype D5 [31,32]. Despite the relative genetic stability, our phylogenetic analysis showed a rapid turnover of D5 lineages since 2014, shifting from lineage 4 dominating in 2014–2018 in France and Spain to newly emerging lineages 1 to 3 dominating in 2022 and 2023. This rapid shift in dominance, together with the rapid disappearance of previous lineages (in our study, all E11 lineage 4 strains are from 2019 and since then no other E11 strains have been observed in this lineage), is a typical pattern observed for other EV types [33,34]. Interestingly, all the E11 strains of new lineage 1 were restricted geographically in western and southern parts of Europe (France, Germany, Italy, Spain and the UK), while lineage 2 strains reached a wider geographical range including China between 2018 and 2023. This demonstrates that a single variant may be rapidly transmitted over a very broad geographic area in a short time [29]. Enhanced molecular surveillance within and outside Europe is needed to determine the extension of the new lineage 1 E11 strains.

Our findings detected three novel recombinant forms, none of which clustered with the new D5 lineage 1. Recombination is a well-known mechanism in EV evolution. It allows the exchange of genomic regions between cocirculating viruses, impacting viral adaptability, escape from the host immune response and pathogenesis and contributes to the emergence of new variants with a potentially greater virulence and clinical burden [35]. Recombination events appeared to occur almost entirely outside of the capsid-encoding region used for genotyping. Therefore, obtaining the full genome is crucial for detecting these events [35]. In this study, the full-length genome was successfully obtained for 10 E11 strains. For the majority of fully-sequenced strains in the present study, other E11 genomes with similar recombinant genomic organisation have been detected previously and are publicly available [6,9]. Interestingly, when comparing the complete genome of strains obtained by our study with similar sequences in GenBank, we observed that SPA1 (2023), SPA7 (2022) and SPA4/SPA6 (2023) presented a mosaic organisation with no previously detected or reported homologous sequences. The finding of a relatively high number (n = 3) of new E11 recombinant forms during a 5-year study period would suggest low-level circulation of these recombinants, or their circulation in a restricted geographical area (Spain) and therefore not captured until now in this study, or a lack of sustained global surveillance for E11 using complete genomes. It may also be possible that the recombination event leading to the circulation of these novel recombinant viruses was recent. Alternatively, they may have been circulating causing mild disease and therefore not captured by surveillance in the EVL which primarily receives samples from hospitalised cases. Interestingly, two of the three novel recombinant E11 viruses were associated with severe disease prompting ICU admission (SPA1 was associated with fatal sepsis with liver failure and SPA7 with severe meningoencephalitis). This emphasises the need to strengthen E11 genomic surveillance to monitor whether these or other new recombinant forms are causing more severe E11 infections in other European countries beyond E11 strains in the new lineage 1.

A disease diagnosed as of E11 aetiology may actually be a coinfection, or entirely caused by another pathogen and E11 was present only as asymptomatic infection. Therefore, a metagenomic approach is warranted when investigating E11 aetiologies. In this study, by using untargeted metagenomics, coinfecting viruses were found in four of 10 E11-positive samples. Although coinfecting viruses were found with much less deep coverage and fewer viral reads, they cannot be excluded as contributing to severe outcomes. For instance, in one sample, we confirmed coinfection by conventional PCR for HPeV-A1, a virus usually affecting neonates and can occasionally lead to sepsis, meningitis or other neurologic manifestations, or even death [36]. In three other samples, human bocavirus and EV-D68 were identified as potential coinfecting viruses. Regarding EV-D68, PCR confirmation was not possible. One possible explanation is that if the viral genome was fragmented, the shorter fragment obtained by mNGS compared with the expected PCR amplification product [15], may have hindered proper primer binding, resulting in a negative PCR result. Another possibility is the low viral load of EV-D68 in CSF samples. It is widely recognised that EV-D68 is rarely detected in CSF during clinical presentation [15,37,38], rendering such samples less optimal for virus detection. Furthermore, the SPA7 sample was collected in 2022, a year when EV-D68 was the predominant enterovirus in Spain, with E11 ranking as the fifth most prevalent (unpublished data). Given their substantial prevalence, coinfection with these two viruses would not be unexpected. Human bocaviruses are frequently associated with respiratory tract infections but can also be associated with encephalitis [39]. Overall coinfections show the complexity involved in reaching a correct diagnosis.

Our work has several limitations. This study focuses only on virome analysis. However, it is important to note that secondary bacterial infections are a common complication associated with viral infections [40]. Another limitation is that burden of severe cases may be under-represented as clinical information is not consistently provided by hospitals when submitting samples. To overcome this limitation, an active search for clinical features has been requested of doctors with a response rate of ca 50%, which provided clinical information in almost 60% of neonatal E11 infections included in this study. Improving the recording of clinical information such as patient clinical symptoms, underlying diseases and ICU admission when requesting sample testing would improve the understanding between E11 infection and severe complications.

## Conclusion

Data presented in this study highlight the value of extensive molecular, genomic and clinical investigations to fully describe E11 infection and evolution in the context of a European alert. While new lineage 1 has been circulating in Spain since 2022, it has not been associated with more severe disease in neonates. Thoroughly investigating potential (co)infections is crucial, as they may contribute to severe or fatal outcomes of E11 infections.

## Supplementary Data

1116000 Supplement
