# Supplementary material for: Enhanced echovirus 11 genomic surveillance in neonatal infections in Spain following a European alert reveals new recombinant forms linked to severe cases, 2019 to 2023
Source: Euro Surveill. 2024 Oct 31;29(44):2400221. doi: 10.2807/1560-7917.ES.2024.29.44.2400221 (PMC11528903; doi:10.2807/1560-7917.ES.2024.29.44.2400221)
Supplement: Supplement [file 24-00221_FERNANDEZ_GARCIA_Supplement.pdf]

Supplementary material

This supplementary material is hosted by *Eurosurveillance* as supporting information alongside the article 'Enhanced echovirus 11 genomic surveillance in neonatal infections in Spain following a European alert reveals new recombinant forms linked to severe cases, 2019 to 2023, on behalf of the authors, who remain responsible for the accuracy and appropriateness of the content. The same standards for ethics, copyright, attributions and permissions as for the article apply. Supplements are not edited by *Eurosurveillance* and the journal is not responsible for the maintenance of any links or email addresses provided therein.

Table of contents

Methods

References

Figures and tables legends

Methods

Metagenomic next-generation sequencing

RNA was extracted from samples using the Quick RNA viral kit (Zymo). Samples were dual indexed during the library preparation conducted using the NEBNext Ultra II Directional RNA Library Prep Kit for Illumina with NEBNext Multiplex Oligos (New England BioLabs Inc., USA) from a total concentration of 5 ng of RNA quantified using QuantiFluor RNA System (E3310, Promega). Target enrichment was performed by hybrid-capture using the Twist Comprehensive Viral Research Panel v2 (Twist Biosciences, San Francisco, CA) that covers reference sequences for 3153 viruses, including 15,488 different strains. Hybrid-capture libraries were combined equally by mass into capture pools (average 5-plex). Twist post-capture library pools were PCR amplified 16 cycles, then quantified by QuantiFluor ONE dsDNA System (Promega, Madison, USA) on the Quantus Fluorometer (Promega) and finally quality verified for sequencing with the Bioanalyzer High Sensitivity DNA Analysis System (Agilent Technologies, Inc. Santa Clara, USA). Enriched libraries were sequenced using a Nextseq 500 Illumina platform (Illumina Inc. San Diego (CA), USA) with a NextSeq 500/550 High Output Kit v2.5.

Metagenomic next-generation sequencing data analysis for taxonomic classification

Viralrecon pipeline was written in Nextflow (https://www.nextflow.io/) in collaboration between the nf-core (https://nf-co.re/) community and the Bioinformatics Unit of the Institute of Health Carlos III (BU-ISCIII) (https://github.com/BU-ISCIII). In this pipeline, fastq files were first analysed for quality control using FastQC v0.11.9 (http://www.bioinformatics.babraham.ac.uk/projects/fastqc/). Raw reads are first analyzed for quality using FastQC v0.11.9(1) and then trimmed using fastp v.0.20.1(2), where a sliding window quality filtering approach was performed, scanning the read with a 4-base wide sliding window, cutting 3′ and 5′ ends base when the average quality drops below a Qphred33 of 30. Reads shorter than 50 nucleotides and reads with more than 10% of bases with quality under Qphred 30 were removed. Additionally, poly-X sequenced were removed from read ends. To obtain statistics about the host genome content, Kmer-based mapping of the trimmed reads against the GRCh38 NCBI human genome reference was performed with Kraken2 v.2.0.9beta(3). Reads asigned to host's genome were removed for further analyses. SPAdes v3.14.0(4) in metaSPAdes mode was used to perform a de novo assembly of non-host reads. Contigs taxonomic annotation was based on alignment to the NCBI's viral database using BLAST v2.9.0+(5), with default parameters and filtering contigs longer than 200bp and alignments with a contig coverage higher than 70%. Apart from the novo assembly, a reference based-mapping approach also was used to obtain complete viral genomes. Trimmed reads were mapped against Echovirus 11 reference genome (OQ969170) using Bowtie2 v.2.4.4(6). Picard v.2.26.10 (https://github.com/broadinstitute/picard) and SAMtools v.1.14(7) were used to sort the aligned BAM files and generate genome mapping statistics.

References

1. Andrews, S. (2010). FastQC: A Quality Control Tool for High Throughput Sequence Data [Online]. Available online at: http://www.bioinformatics.babraham.ac.uk/projects/fastqc/.
2. Chen S, Zhou Y, Chen Y, Gu J. fastp: an ultra-fast all-in-one FASTQ preprocessor | Bioinformatics | Oxford Academic. Bioinformatics. 2018;
3. Wood DE, Lu J, Langmead B. Improved metagenomic analysis with Kraken 2. Genome Biology. 2019;
4. Nurk S, Meleshko D, Korobeynikov A, Pevzner PA. metaSPAdes: a new versatile metagenomic assembler. Genome research [Internet]. 2017;27(5):824–34. Available from: http://www.ncbi.nlm.nih.gov/pubmed/28298430%0Ahttp://www.pubmedcentral.nih.gov/articlerender.fcgi?artid=PMC5411777
5. 4. BLAST® Command Line Applications User Manual [Internet]. Bethesda (MD): National Center for Biotechnology Information (US); 2008-. Available from: https://www.ncbi.nlm.nih.gov/books/NBK279690/.
6. Langmead B, Salzberg SL. Fast gapped-read alignment with Bowtie 2. Nature Methods. 2012;
7. Li H, Handsaker B, Wysoker A, Fennell T, Ruan J, Homer N, et al. The Sequence Alignment/Map format and SAMtools. Bioinformatics. 2009;25(16):2078–9.
8. Kalantar KL, Carvalho T, De Bourcy CFA, Dimitrov B, Dingle G, Egger R, et al. IDseq-An open source cloud-based pipeline and analysis service for metagenomic pathogen detection and monitoring. GigaScience. 2021;

Figures and tables

Supplementary Figure S1. Non-collapsed phylogenetic tree of partial VP1 coding sequences of Echovirus 11 study strains and sequences from previously described Echovirus 11 strains extracted from GenBank (n=134). Sequences obtained in this study are indicated by coloured circles with colour according to year of sampling. The neighbor-joining tree was constructed by using MEGA 10.0. Phylogenetic analysis was inferred using the Neighbor-Joining method on the Kimura-2 parameter model. Support for tree nodes was assessed by bootstrap values based on 1000 replications. Numbers on nodes indicate the bootstrap support of node (>80). Scale bar represents nt substitutions per site. The prototype Echovirus 11 sequence was introduced as outgroup (Acc nº. X80059).

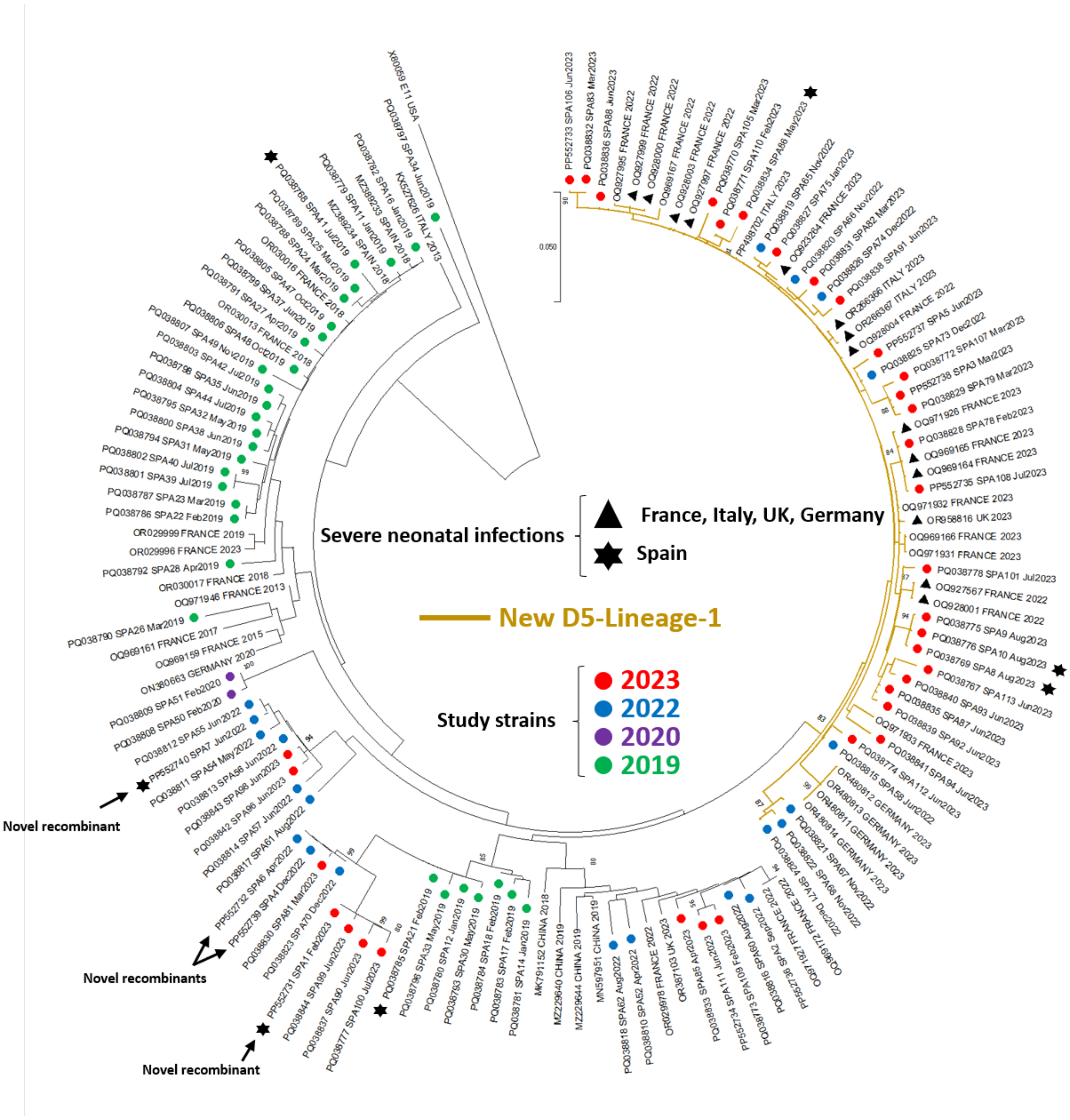

Supplementary Figure S2. Phylogenetic analysis of complete genome sequences, Echovirus 11. Phylogenetic analysis was inferred using the Neighbor-Joining method.

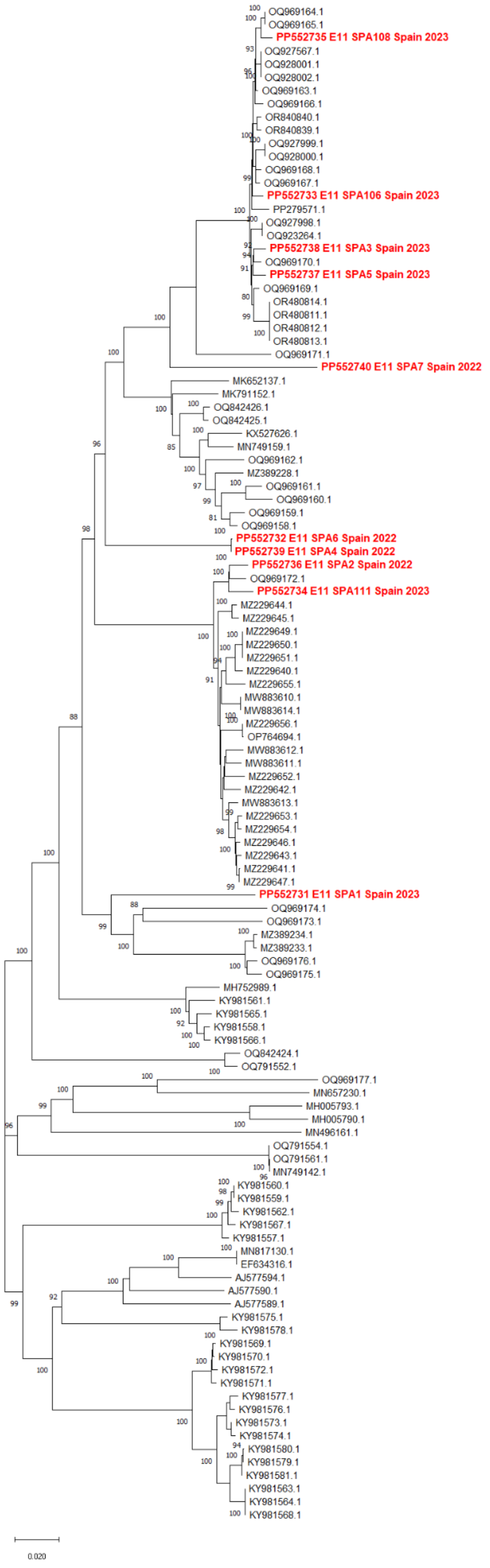

Supplementary Figure S3. Non-collapsed phylogenetic tree of complete VP1 coding sequences of Echovirus 11 corresponding to figure 3. Phylogenetic analysis was inferred using the Neighbor-Joining method.

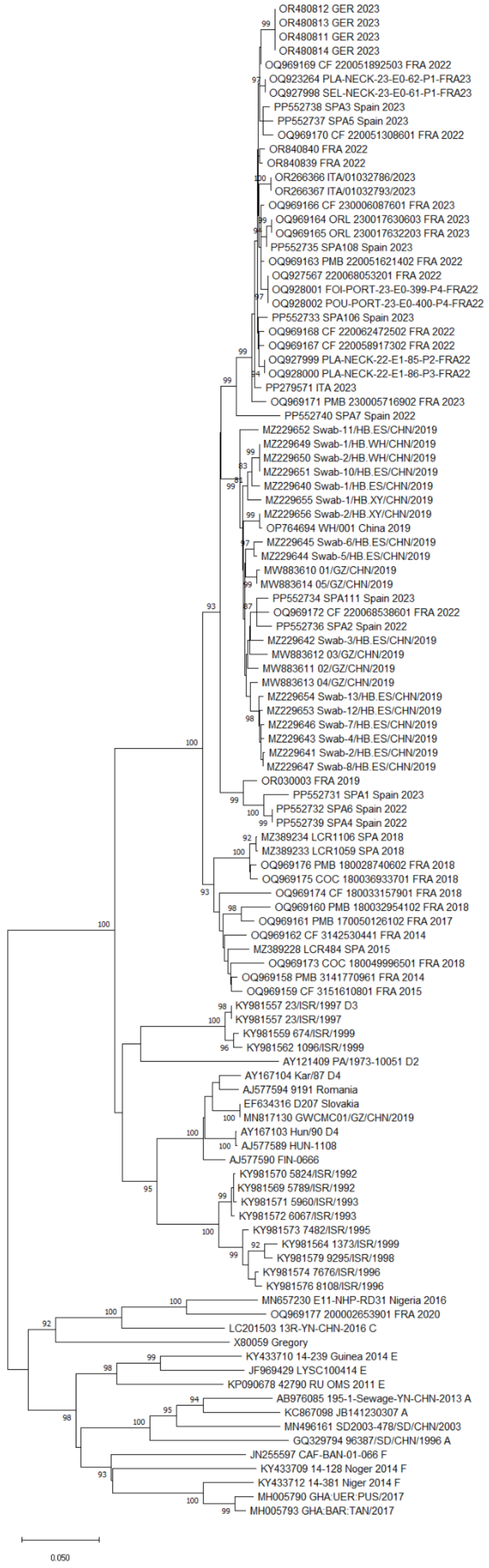

Supplementary Figure S4. Phylogenetic trees of complete P1 (A), P2 (B) and P3 (C) coding sequences of Echovirus 11 study strains, sequences from previously described Echovirus 11 strains extracted from GenBank and representative fully sequenced EV-B reference genomes available in GenBank. Sequences obtained in this study are indicated in red. Sequences of closely related types are indicated in blue. These sequences were those with highest score when using the P2 and P3 genomic region of study strains novel recombinant forms as queries for BLASTn (Supplementary Table S2). The prototype E11 strain (X80059) is indicated in green. Phylogenetic analyses were inferred using the Neighbor-Joining method. Numbers on nodes indicate the bootstrap support of node (>80). Scale bar represents nt substitutions per site.

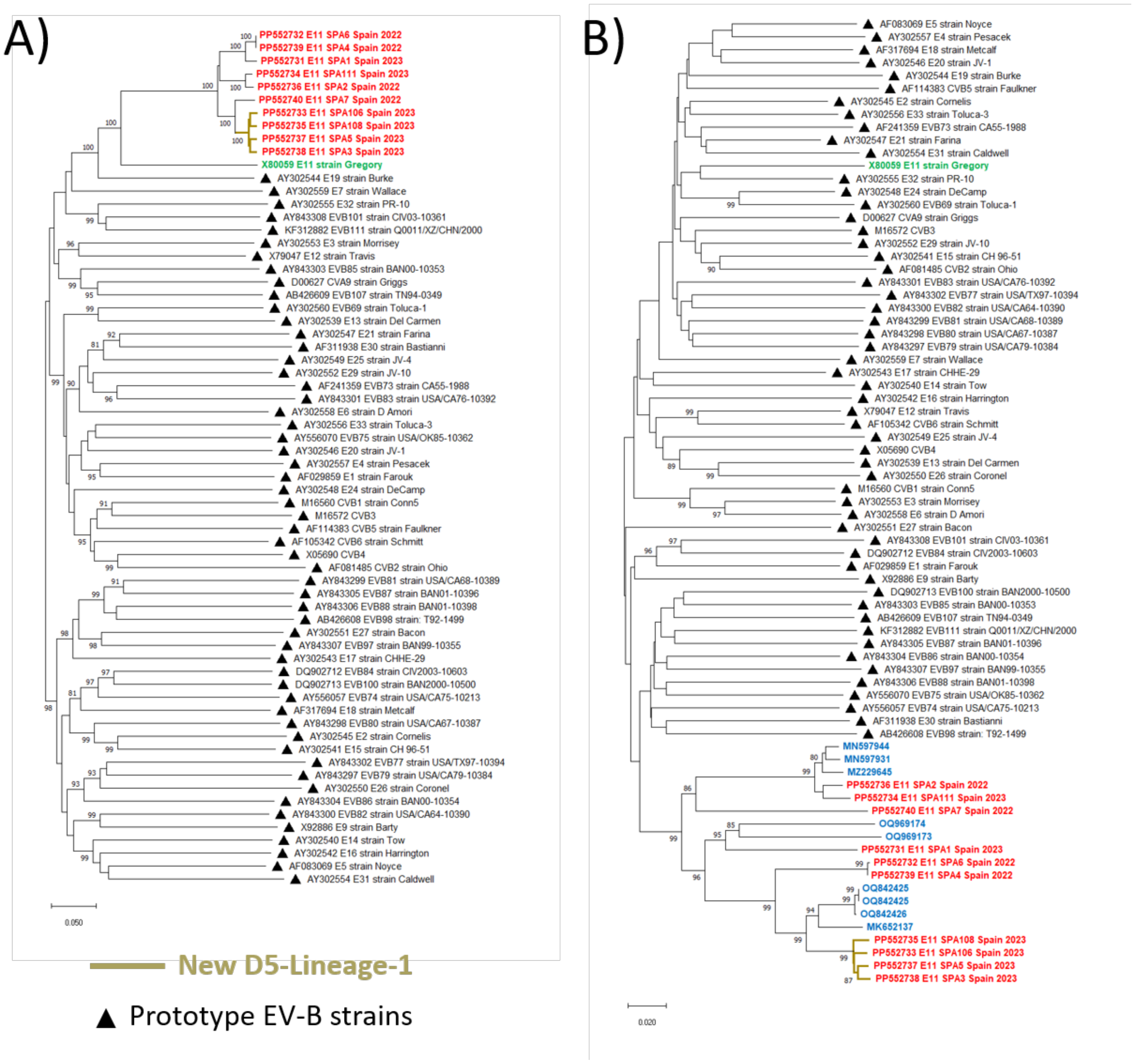

C)

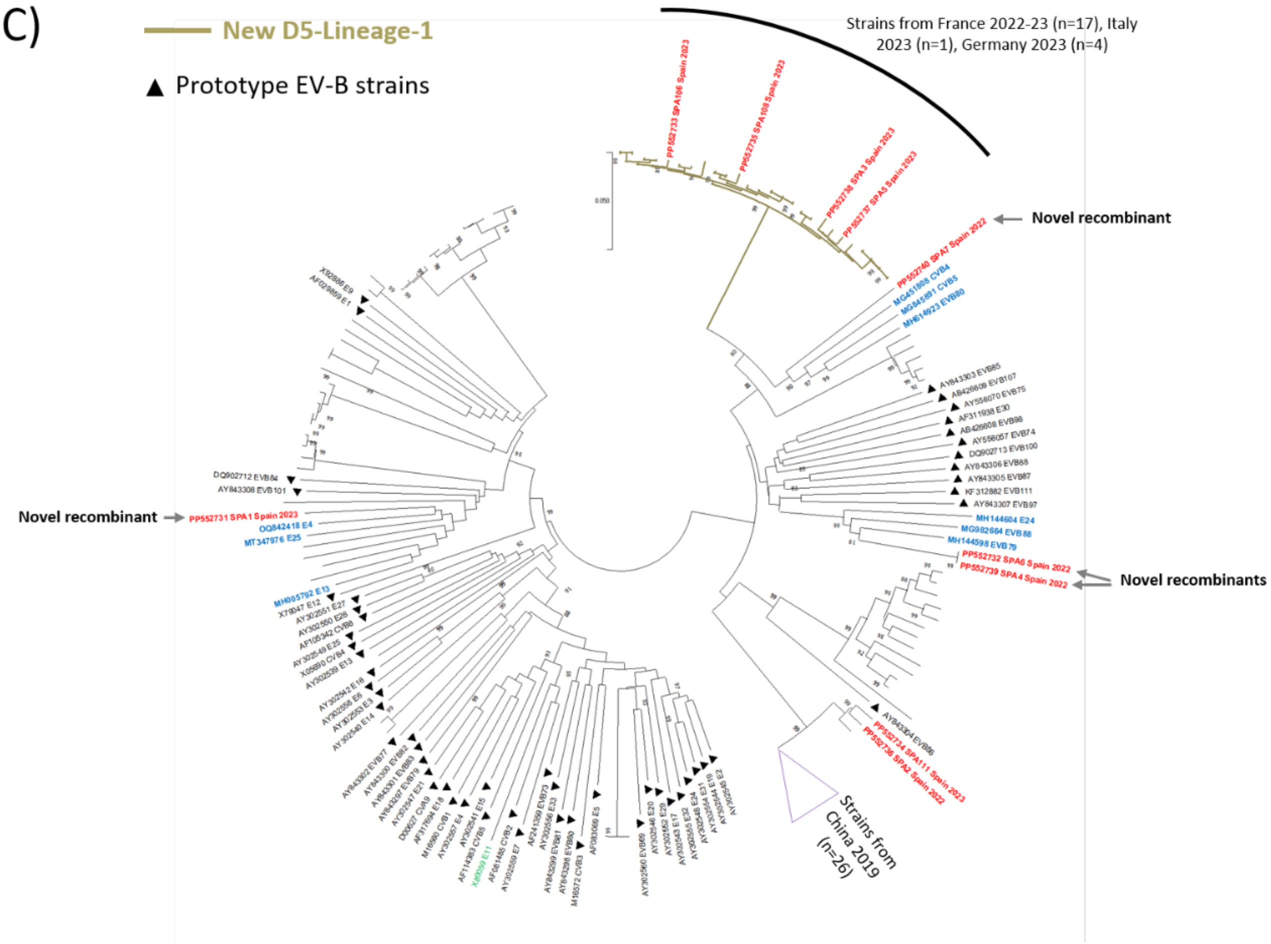

New D5-Lineage-1
▲ Prototype EV-B strains
Strains from France 2022-23 (n=17), Italy 2023 (n=1), Germany 2023 (n=4)
Novel recombinant
Novel recombinant
Novel recombinants
Strains from China 2019 (n=26)

Supplementary Figure S5. Plot of similarity of whole-genome nucleotide sequences of study strains SPA1, SPA7 and SPA4-6 and a selection of Echovirus 11 strains from NCBI corresponding to new lineage-1.

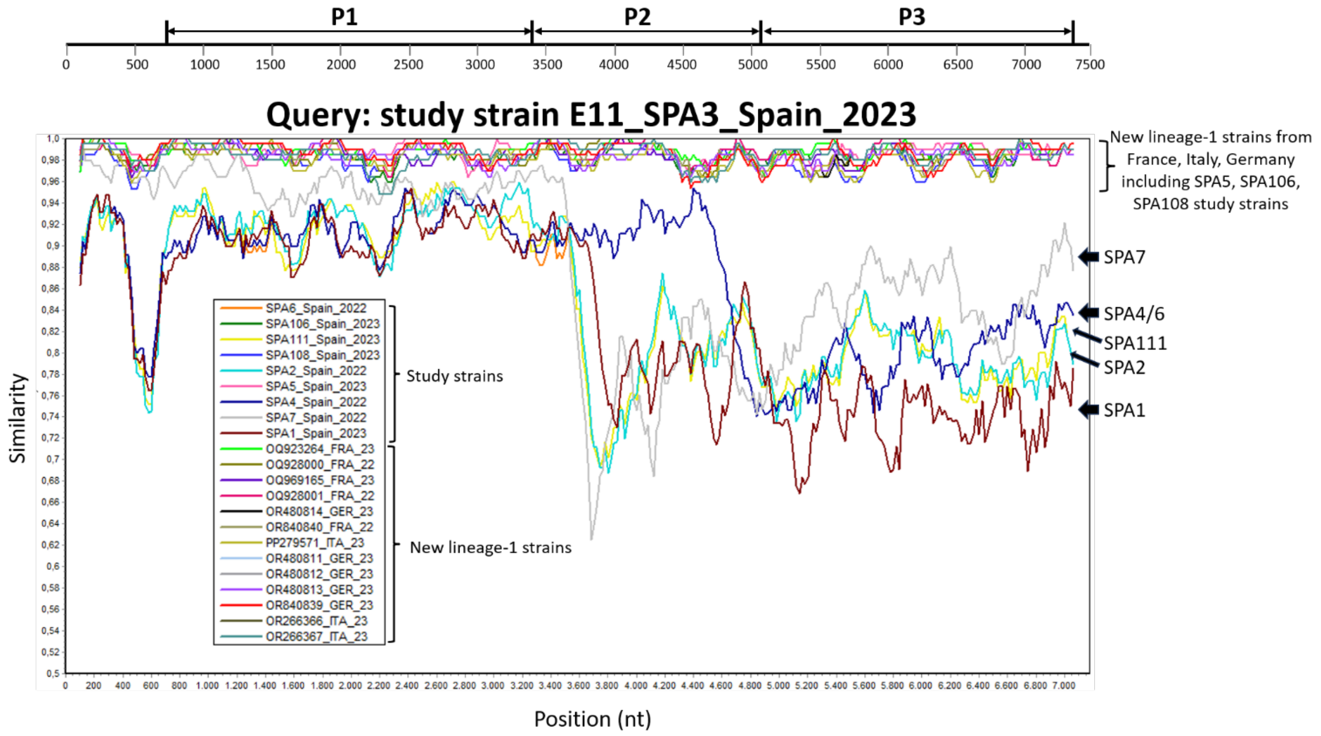

Supplementary Table S1. Metagenomic sequencing results

Sample strain	Virus hit*	Accession Numbers	Total number reads	% viral reads mapped to viral contigs	% host reads	Median coverage depth	Genome coverage %	Nt. length	rPM
SPA1	E11	PP552731	454716	12.2	56.9	293	>99	7377	303070.4
SPA2	E11	PP552736	771298	0.61	0	47	>99	7376	24871.9
SPA3	E11	PP552738	32592	78.63	2.9	237	>99	7336	599381.6
	HBoV2	PQ039526		0		13	4.5	232	577
SPA4	E11	PP552739	22474924	75.1	0.1	108861	>99	7378	742392
	PeVA1	PQ066000		0.01		16	67.1	4896	41
SPA5	E11	PP552737	1052874	53.94	0	7212	>99	7378	436007.7
SPA6	E11	PP552732	88708	0.69	95.7	70	>99	7377	7739.5
SPA7	E11	PP552740	54000	37.07	27.0	189	>99	7379	365088
	EVD68	PQ039529		5.8		10	4	272	441
SPA106	E11	PP552733	3579240	89.06	10.6	38651	>99	7357	663391.2
SPA108	E11	PP552735	434858	15.96	80.5	869	>99	7356	82786.2
SPA111	E11	PP552734	1053266	39.31	15.6	3068	>99	7354	584628.5

	HBoV1	PQ039527		0		38	5.5	286	116

EV: enterovirus; E11: Echovirus 11; HBoV: Human Bocavirus; PeV: Parechovirus; rPM: Number of reads aligning to the taxon in the NCBI NR/NT database, per million reads sequenced; Virus Hit: viral taxon with the highest abundance of reads matching to that taxon in the NT and NR database while being less prevalent in control samples selected for the applied background model. Those taxa in the top score have more than 1 reads per million matching the NT and NR databases (NT rPM > 1 and NR rPM > 1) and are found in higher abundance in samples than in negative controls (NT Z score > 1 and NR Z score > 1) (8)

Supplementary Table S2. Closely related sequences available in the GenBank database selected using BLAST.

Genomic region	Sample Nº	Closely related type	Strain	Country	Year	Accesion Number	Query cover	Per. Ident
P3	SPA7	CVB4	Env_2017_Jan_CV-B4	UK	2017	MG451808	100%	88.18%
		EV-B80	HT-TSLH64F/XJ	China	2011	MH614923	100%	88.01%
		CVB5	B5L060815	Switzerland	2015	MG845891	100%	88.01%
	SPA1	E4	E4/USA/8G9/2011	USA	2011	OQ842418	100%	88.68%
		E25	USA/2016-19521	USA	2016	MT347976	99%	87.34%
		E13	GHA:VOL:KRN/2017	Ghana	2017	MH005792	100%	87.13%
	SPA4/6	EV-B79	17-2255-1_E79	India	2017	MH144598	100%	91.27%
		EV-B88	NIV-17-3150-2	India	2017	MG982664	100%	89.52%
		E24	13-2053-1_E24	India	2013	MH144604	100%	88.3%
P2	SPA7	E11	Swab-6/HB.ES/	China	2019	MZ229645	100%	85.66%
		E11	Sewage10-1-1/GD.GZ/	China	2019	MN597944	100%	85.53%
		E11	Sewage8-2-1/GD.GZ/	China	2019	MN597931	100%	85.55%
	SPA1	E11	CF_180033157901	France	2018	OQ969174	99%	86.85%
		E11	COC_180049996501	France	2018	OQ969173	99%	86.54%
		E11	E11/USA/11M9/2014	USA	2014	OQ842425	99%	86.58%
	SPA4/6	E11	E11/USA/11M9/2014	USA	2014	OQ842425	100%	91.98%
		E11	USA/2018-23090	USA	2018	MK652137	100%	91.87%
		E11	E11/USA/11N9/2014	USA	2014	OQ842426	100%	91.75%
